# MARVEL Analysis of High‐Resolution Rovibrational Spectra of ^16^O^13^C^18^O

**DOI:** 10.1002/jcc.27541

**Published:** 2024-12-19

**Authors:** Ala'a A. A. Azzam, Jonathan Tennyson, Sergei N. Yurchenko, Tibor Furtenbacher, Attila G. Császár

**Affiliations:** ^1^ Department of Physics The University of Jordan Amman Jordan; ^2^ Department of Physics and Astronomy University College London London UK; ^3^ HUN‐REN–ELTE Complex Chemical Systems Research Group Budapest Hungary; ^4^ Institute of Chemistry ELTE Eötvös Loránd University Budapest Hungary

**Keywords:** ^16^O^13^C^18^O, carbon dioxide, high‐resolution spectroscopy, line positions, MARVEL analysis, rovibrational energy levels

## Abstract

A large set of validated experimental transitions and empirical rovibrational energy levels are reported for the fifth most abundant carbon dioxide isotopologue, ^16^O^13^C^18^O (in a shorthand notation, 638). Validation of the transitions and determination of the empirical energy levels are based on a compiled and carefully checked dataset, collected from 35 literature sources, containing 12 348/7432 measured/unique lines in the wavenumber range of 578–9318 cm^−1^. The MARVEL (Measured Active Rotational‐Vibrational Energy Levels) protocol, built upon the theory of spectroscopic networks, not only validates the vast majority of the measured transitions, but also yields 3975 empirical rovibrational energy levels, with uncertainty estimates compliant with the experimental uncertainties of the transitions.

## Graphical abstract

12348 measured transitions in the wavenumber range 578–9318 cm^−1^ for carbon dioxide (isotopologue 638) were used° to calculating 3975 empirical rovibrational energy levels with J° up to 82.

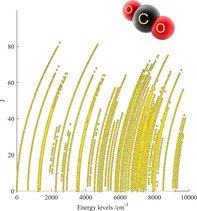

## 1 Introduction

Recently, we decided to construct an extensive database of empirical rovibrational energy levels, based on spectral features measured under high resolution, for all the isotopologues of carbon dioxide involving the ^12^C, ^13^C, ^16^O, ^17^O, and ^18^O isotopes. The present contribution extends the list of CO_2_ isotopologues treated by us, ^16^O^13^C^16^O (636, according to a shorthand notation following HITRAN [1] parlance) [2], ^16^O^12^C^18^O (628) [3], ^18^O^12^C^18^O (828) [4], ^18^O^13^C^18^O (838) [4], and ^17^O^12^C^18^O (728) [4]. Investigation of the parent isotopologue, ^16^O^12^C^16^O (626), is also under way by our laboratories [5]. With a natural abundance of 0.004 434%, the carbon dioxide isotopologue ^16^O^13^C^18^O (638) is the fifth most abundant isotopologue of CO_2_ [1]. Similar to the other projects, the empirical rovibrational energy levels of 638 are determined in this study using the MARVEL (Measured Active Rotational‐Vibrational Energy Levels) procedure [6, 7, 8, 9], built upon the theory of spectroscopic networks [10, 11], and the related code MARVEL 4.0.

The importance of the understanding of the energy‐level structure and the high‐resolution rovibrational spectra of CO_2_ has been emphasized a number of times [1, 2, 3, 4, 12, 13]. For example, carbon dioxide (a) is one of the most important constituents of a large number of planetary atmospheres, including that of Earth [14], where it has a significant contribution to the man‐made greenhouse effect, (b) has an especially significant contribution to the radiative balance of the atmospheres of our neighboring planets Mars and Venus, and (c) has been used to establish the ratio of carbon isotopologues in the atmospheres of exoplanets [15]. Research on the high‐resolution spectroscopy of carbon‐dioxide isotopologues is very important as it supports such studies.

As to the energy‐level structure of carbon dioxide, an especially restrictive rule applies for the symmetric CO_2_ isotopologues: half of the rotational states are forbidden by the Pauli principle. Relaxation of this rule in asymmetric isotopologues increases the importance of these trace species, as all rotational states are allowed, leading to much denser spectra with lines displaced from those of the parent isotopologue. Thus, the many more allowed rovibrational transitions of 638 make their relative contribution to the detection of this molecule significantly more important than they would be based on the relative abundance of this minor isotopologue.

## 2 Methodological Details

### 2.1 Marvel

The MARVEL procedure [6, 7, 8, 9] includes the construction of a spectroscopic network (SN) [10] from observed spectral line center positions, whereby each energy level (quantum state) serves as a vertex of the SN, and pairs of vertices are connected with the observed transitions forming the edges of the network. Under ideal circumstances, the larger the number of measured and collected spectral lines, the more empirical energy levels can be determined, or at least they are determined with higher accuracy. However, due to the limited number of experimental data available, one usually arrives at a fragmented SN, resulting in a principal component, where all the vertices are linked to the ground state, and a number of isolated components, which need to be connected to the principal one using accurately‐known calculated or semi‐empirical lines. For each spectral line, uncertainties, as well as unique labels, containing quantum numbers characterizing the upper and lower states, must be included in the input file for MARVEL. After executing the MARVEL procedure, the output file contains empirical energy levels, supplemented with educated estimates of their uncertainties. Here the bootstrap method implemented in MARVEL 4.0 [16] is used to determine the final uncertainties of the energy levels.

Inconsistencies, that is, line center positions that significantly deviate from the majority of the data provided are detected straightforwardly during a MARVEL run. This feature proves invaluable for identifying issues with the experimental data, whether stemming from user mistakes during data collection and analysis or from misassignments of the experimental features. The MARVEL input file was regularly tested not only for incorrectly labelled transitions, but also for those which do not obey well‐established selection rules.

### 2.2 Notation and Quantum Numbers

In its ground electronic state, CO_2_ is a linear molecule; it has three fundamental vibrational modes, usually denoted as $\nu_{1}$ (symmetric stretch mode), $\nu_{2}$ (bend mode), and $\nu_{3}$ (antisymmetric stretch mode), associated with the vibrational quantum numbers $v_{i},i=$ 1, 2, and 3, respectively. The doubly‐degenerate $\nu_{2}$ bending mode is characterized by an angular momentum, traditionally described by the quantum number 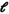. Due to Fermi‐resonance interactions between the states ($v_{1},{\left(v_{2}+2\right)}^{\ell },v_{3}$) and ($v_{1}+1,v_{2}^{\ell },v_{3}$), the vibrational states of CO_2_ are customarily denoted by the so‐called AFGL (Air Force Geophysics Laboratory) notation [17, 18, 19]. Using the AFGL notation, the vibrational energy levels are labeled as ($v_{1},v_{2},\ell ,v_{3},r$), where r is the ranking index for states in Fermi resonance. The polyad number P, defined as $P=2v_{1}+v_{2}+3v_{3}$, collects the interacting states in blocks. P is not a quantum number, but behaves like one. None of these quantum numbers are “good” ones; thus, there are no strict selection rules for them.

For the purposes of a MARVEL analysis, each rovibrational state of CO_2_ must be uniquely characterized by a set of descriptors. For 638, the set of descriptors used is (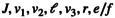). The only good quantum number among the set of descriptors is J, associated with the rotational angular momentum. Allowed rovibrational transitions are governed by the selection rules ΔJ=±1 and ΔJ=0. Parity is also considered to be exact. Thus, the final descriptor we attach to a rovibrational energy level is the rotationless parity p, which is denoted here as either ‘e’ or ‘f’ [20]. The dipole selection rule for this parity is as follows: if ΔJ=0, then e↔f, while if ΔJ=±1, then e↔e or f↔f. The upper and lower states involved in a transition are denoted by 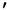 and 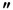, respectively. The P, R, and Q transitions are specified using the lower‐state rotational quantum number, $J^{\prime \prime }$.

### 2.3 Experimental Studies of Line Positions

Many rovibrational transitions for the 638 isotopologue of carbon dioxide have been detected experimentally [21, 22, 23, 24, 25, 26, 27, 28, 29, 30, 31, 32, 33, 34, 35, 36, 37, 38, 39, 40, 41, 42, 43, 44, 45, 46, 47, 48, 49, 50, 51, 52, 53, 54, 55, 56]. The the experimental source of rovibrational transitions collected are summarized in Table 1. In total, 12 362 experimentally measured transitions have been collected from 35 literature sources, all of them are within the range 578−9318 cm^−1^. Of all the experimentally measured transitions, only 7432 are unique. During the MARVEL analysis, 21 transitions had to be removed from the spectroscopic network (SN) set up. They are listed with negative wavenumber entries in the transition file given in the Supplementary Information to this paper.

**TABLE 1:** Experimental sources of rovibrational transitions of ^16^O^13^C^18^O used during the MARVEL analysis of this study, and certain characteristics of these sources.

Source	Range/cm^−1^	A/V/D 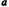	$CSU^{b}$	$MSU^{c}$
85Jolma [21]	578.71–722.69	335/335/0	5.0×10^−3^	5.0×10^−3^
98DeBeSmRi [22]	1211.71–1375.84	186/186/0	2.0×10^−4^	3.1×10^−4^
85Toth [23]	1224.55–1369.31	46/46/0	5.0×10^−3^	5.0×10^−3^
84RiBeDeFe [24]	1980.52–2614.51	39/39/0	1.0×10^−3^	1.1×10^−3^
85RiBeDe [25]	1980.52–2006.68	20/20/0	1.0×10^−2^	1.0×10^−2^
86EsSaRoVa [26]	2154.83–21812.84	796/796/0	9.0×10^−4^	1.0×10^−3^
83EsRo [27]	2157.47–2311.15	181/181/0	6.9×10^−4^	8.1×10^−4^
82EsHuSaVa [28]	2162.66–2311.15	176/176/0	1.0×10^−3^	1.1×10^−3^
86EsRo [29]	2165.05–2286.94	187/187/0	5.0×10^−4^	9.8×10^−4^
86BaRo [30]	2192.01–2305.11	163/163/0	1.0×10^−3^	1.0×10^−3^
12LyKaJaLu [31]	2194.32–4838.49	500/500/0	1.0×10^−3^	1.3×10^−3^
78BaLiDeRa [32]	2209.88–2298.64	86/86/0	3.0×10^−3^	3.3×10^−3^
68ObRaHaMc_PENN‐STATE [33]	2235.89–2287.09	69/61/8	2.8×10^−2^	3.1×10^−2^
68ObRaHaMc_OHIO‐STATE [33]	2235.92–2284.24	39/34/5	2.5×10^−2^	3.5×10^−2^
67Hahn [34]	2235.97–2273.03	39/34/5	1.0×10^−3^	7.5×10^−3^
03DeBeSmRi [35]	2240.56–2289.27	48/48/0	2.0×10^−4^	2.4×10^−4^
84DeRiBe [36]	2567.88–2614.51	27/27/0	1.0×10^−3^	1.3×10^−3^
13RoBoAuDr [37]	2682.92–2725.66	56/56/0	7.2×10^−2^	7.2×10^−2^
14BoJaLyTa [38]	3421.04–4679.99	398/398/0	3.0×10^−4^	4.9×10^−4^
08ToMiBrDe [39]	3437.73–3621.29	159/159/0	1.0×10^−3^	1.1×10^−3^
82BaRiSmRa [40]	3453.51–3607.95	79/79/0	5.0×10^−3^	6.2×10^−3^
03DiPeTaTe [41]	4449.06–8024.48	1372/1372/0	1.0×10^−3^	1.5×10^−3^
02Miller [42]	4469.44–4954.20	352/351/1	2.0×10^−5^	1.2×10^−4^
15BoJaLyTa [43]	4682.34–4946.90	103/103/0	4.7×10^−4^	8.5×10^−4^
18KaSiCeMo [44]	5698.72–5851.16	555/555/0	1.0×10^−3^	1.1×10^−3^
18CeKaMoKa [45]	5703.11–5832.29	158/158/0	1.0×10^−3^	1.1×10^−3^
18KaCeMoKa [46]	5703.11–5877.33	513/513/0	1.0×10^−3^	1.1×10^−3^
08PePeCa [47]	5851.97–7035.68	534/534/0	1.0×10^−3^	1.3×10^−3^
14KaCaMoKa [48]	5867.62–6744.16	1375/1375/0	1.0×10^−3^	1.2×10^−3^
06PeKaRoPe [49]	5957.24–6122.25	628/628/0	1.0×10^−3^	1.3×10^−3^
07PeKaRoPe [50]	5967.50–6319.32	41/41/0	1.0×10^−3^	1.0×10^−3^
04DiMaRoPe [51]	6099.92–6744.16	1313/1312/1	1.0×10^−3^	1.6×10^−3^
17KaCaKaTa [52]	6982.58–7917.50	516/515/1	1.0×10^−3^	1.2×10^−3^
10CaSoMoPe [53]	7029.57–7916.90	1044/1044/0	8.0×10^−4^	9.4×10^−4^
14KaKaTaPe [54]	7914.36–8024.24	92/92/0	1.5×10^−3^	1.6×10^−3^
05DiCaBeTa [55]	9153.61–9317.09	123/123/0	2.0×10^−3^	3.5×10^−3^

*Note:* 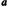 A/V/D = Available/Validated/Deleted transitions (transitions in floating components cannot be validated, but are not deleted). 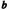 CSU= Average claimed source uncertainty. 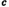 MSU= Average MARVEL suggested uncertainty.

The SN of the experimentally measured transitions of 638 contains four large floating components. These floating components contain 48 rovibrational energy levels (vertices). To connect these floating components to the principal component, we used calculated transitions taken from the Carbon Dioxide Spectroscopic Databank (CDSD‐296) [12]. Altogether 14 CDSD‐296 transitions, with $5\times 10^{-3}$ cm^−1^ uncertainty, were added to the MARVEL database of experimentally measured transitions. The tag of these lines in the input transition file is ‘19CDSD’.

## 3 Results and Discussion

### 3.1 Main Results

**FIGURE 1:**
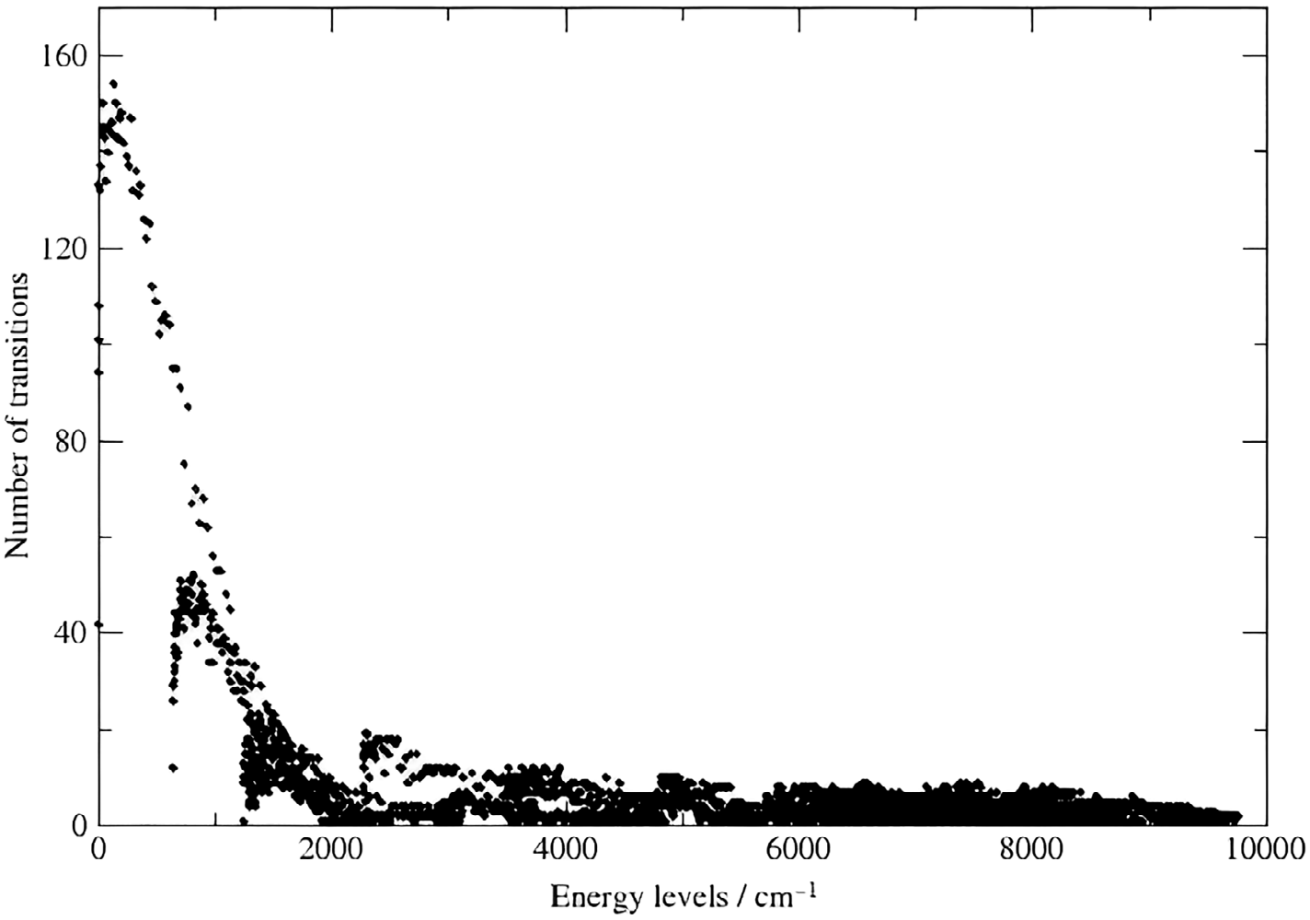
The number of transitions incident to each energy level versus the empirical rovibrational energy level values determined in this study.

Using the MARVEL procedure, 3975 empirical rovibrational energy levels have been obtained for 638, determined by 12 348/7432 measured/unique observed transitions. Figure 1 shows the number of transitions incident to each energy level and their distribution as a function of energy. This figure confirms that the degree distribution of the nodes of spectroscopic networks formed by experimentally measured transitions appear to be heavy‐tailed [57]. The consequences of are as follows: (a) the appearance of “hubs,” that is, a small number of highly interconnected nodes, (b) a connection preference that is, generally disassortative [58], that is the SN's high‐degree vertices preferentially attach to low‐degree ones, (c) considerable robustness and error tolerance, and (d) an “ultra‐small‐world” property, that is the average length of the shortest paths scales as ∼loglog*N*, where *N* is the number of nodes in the SN.

**FIGURE 2:**
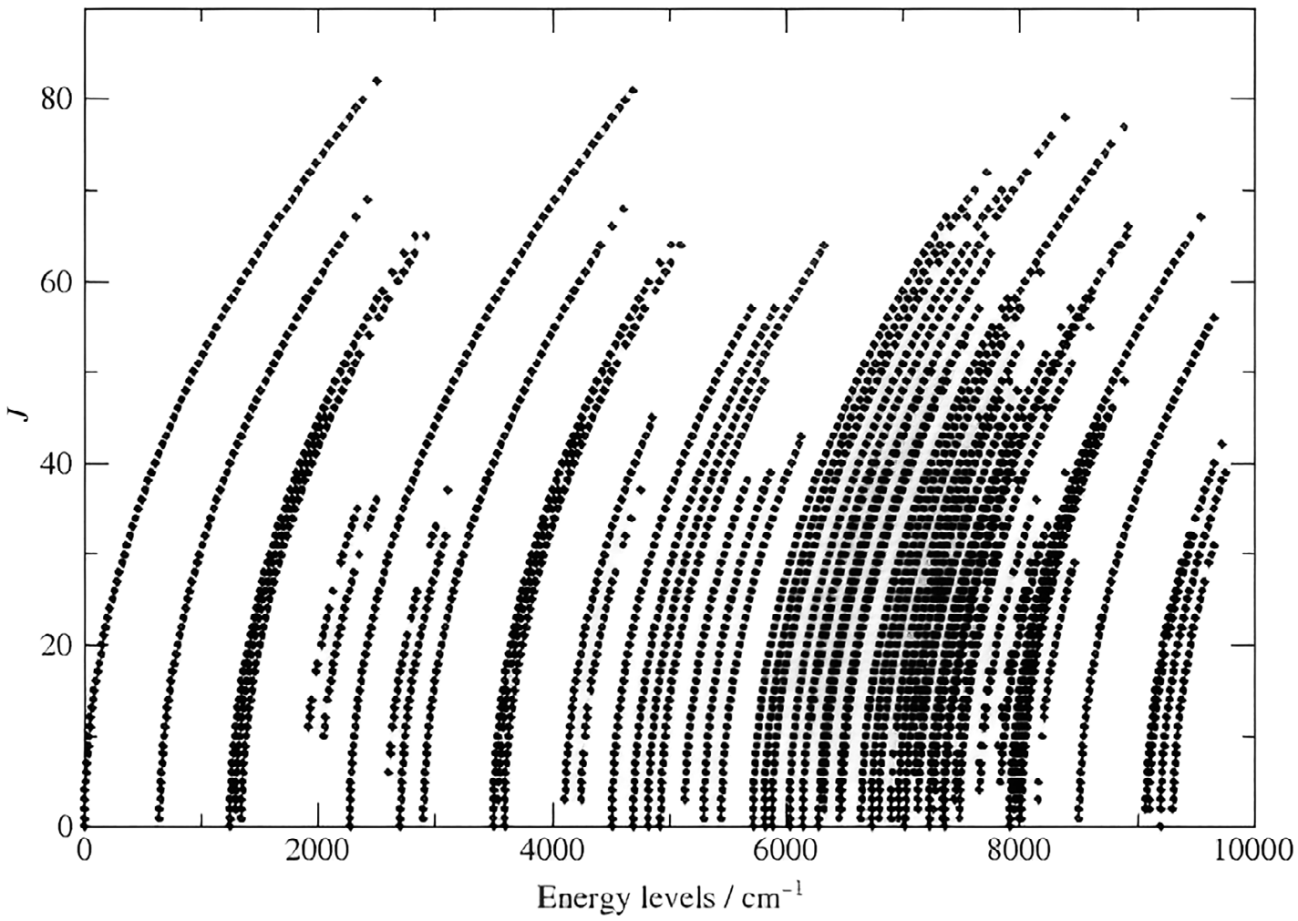
Rotational quantum number J for each calculated energy level versus the empirical energy levels determined for ^16^O^13^C^18^O using the MARVEL procedure.

The empirical rovibrational energy levels determined in this study have J values up to 82, as seen in Figure 2. Each dotted curve in Figure 2 corresponds to a vibrational band, with the dots representing different J values. The energies of the 22 vibrational band origins (VBO), where J=0, are listed in Table 2. The stacks of energy levels clearly show the semirigid character of the CO_2_ molecule. Note also the nice agreement between the MARVEL‐based VBOs determined in this study and those reported in the CDSD‐296 databank [12].

**TABLE 2:** Experimentally determined vibrational band origins of the ^16^O^13^C^18^O molecule obtained in this study (‘MARVEL’) and their comparison with the corresponding entries of the CDSD‐296 (‘CDSD’) dataset [12].

Label	MARVEL energy/cm^−1^	CDSD energy/cm^−1^
0 0 0 0 0 1 e	0.000 0 (0)	0.000 000
0 1 0 0 0 2 e	1244.899 7 (5)	1244.899 743
0 0 0 0 1 1 e	2265.970 9 (11)	2265.971 552
0 2 0 0 0 1 e	2701.956 1 (740)	2701.936 293
0 1 0 0 1 2 e	3490.396 4 (5)	3490.394 853
0 1 0 0 1 1 e	3587.546 8 (5)	3587.548 613
0 0 0 0 2 1 e	4508.746 6 (11)	4508.747 345
0 2 0 0 1 3 e	4692.173 7 (5)	4692.178 083
0 2 0 0 1 2 e	4814.560 1 (5)	4814.561 614
0 2 0 0 1 1 e	4925.020 0 (5)	4925.017 861
0 1 0 0 2 2 e	5712.609 1 (1)	5712.609 317
0 1 0 0 2 1 e	5809.864 4 (30)	5809.861 989
0 3 0 0 1 4 e	5876.594 7 (11)	5876.595 711
0 3 0 0 1 3 e	6026.624 1 (11)	6026.624 885
0 3 0 0 1 2 e	6140.122 5 (11)	6140.122 650
0 3 0 0 1 1 e	6279.488 1 (11)	6279.488 663
0 0 0 0 3 1 e	6728.354 3 (11)	6728.354 758
0 2 0 0 2 2 e	7017.890 5 (11)	7017.891 780
0 4 0 0 1 4 e	7220.735 4 (9)	7220.737 827
0 4 0 0 1 3 e	7351.690 67 (9)	7351.691 716
0 1 0 0 3 2 e	7911.568 8 (9)	7911.569 220
0 2 0 0 3 2 e	9198.188 6 (20)	9198.189 034

**FIGURE 3:**
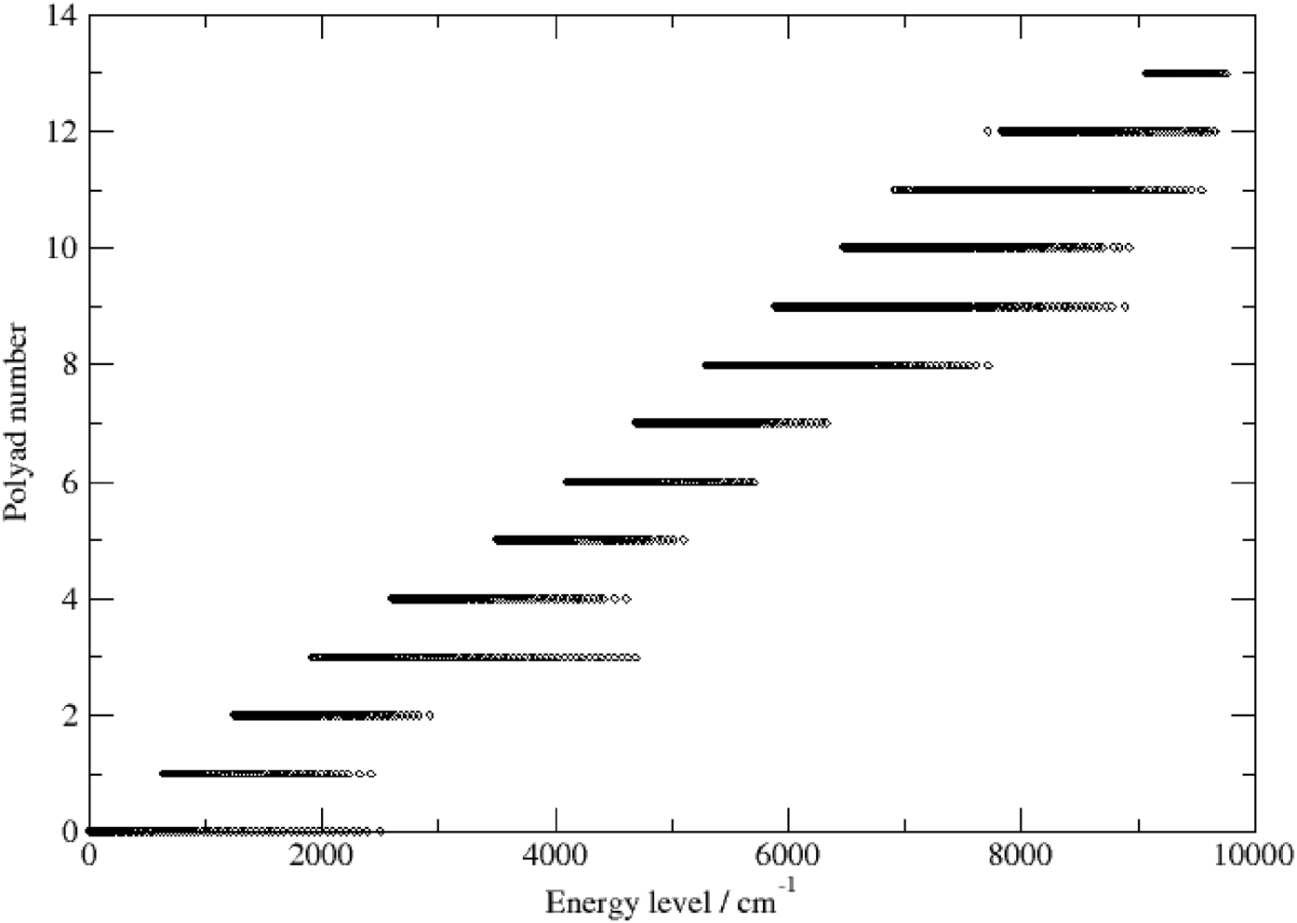
The polyad number, $P=2v_{1}+v_{2}+3v_{3}$, for each empirical energy level versus the energy levels calculated using MARVEL.

Figure 3 shows the polyad numbers, P, for the empirical energy levels of 638 as a function of energy. The values range from 0 up to 13. Note also the nearly linear, stepwise increase of P with the increase of the energy.

**FIGURE 4:**
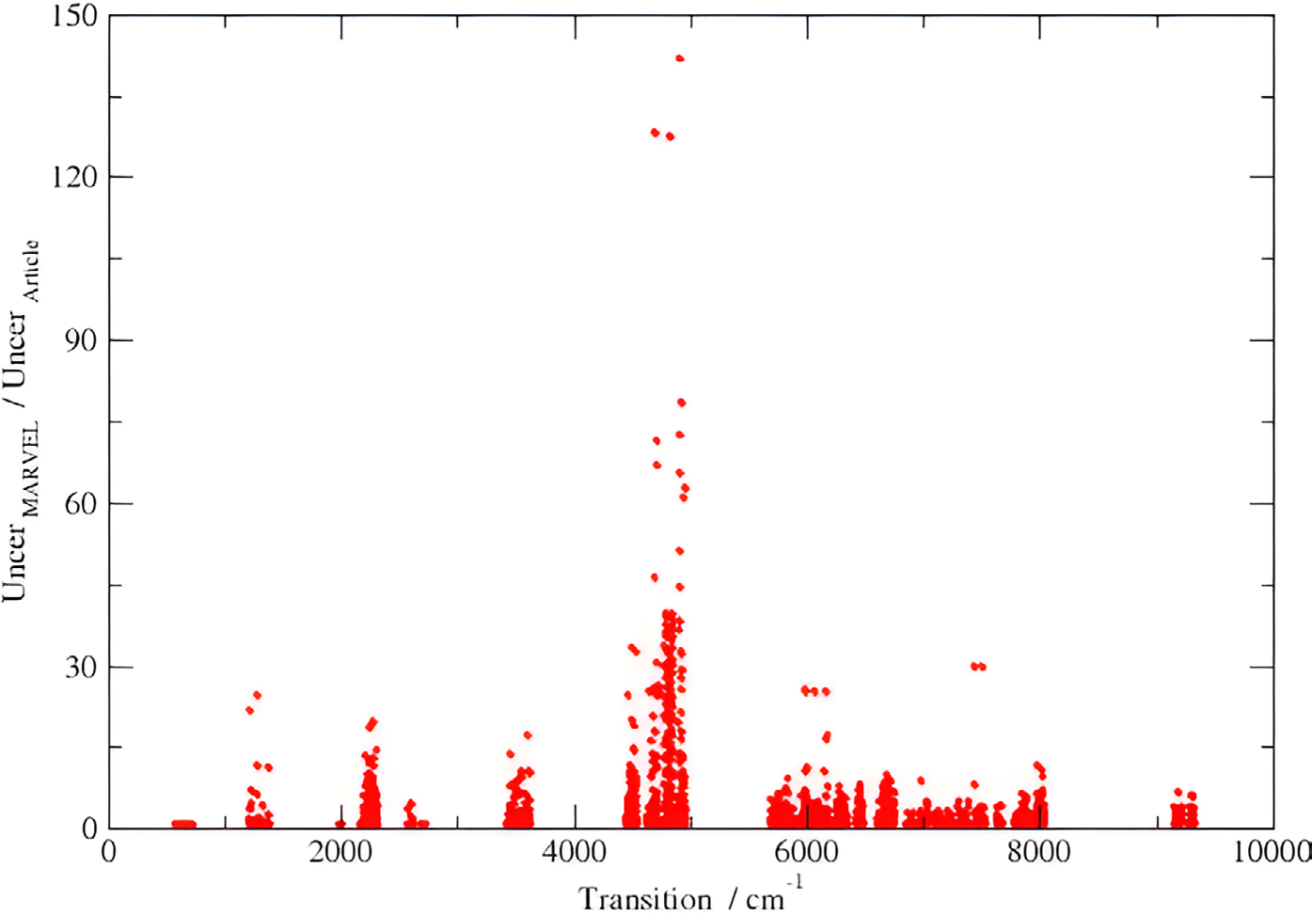
The ratio between the transition uncertainties suggested by MARVEL to the transition uncertainties given by the source articles versus the transition wavenumbers.

Figure 4 shows the ratio between the transition uncertainties suggested by MARVEL and the transition uncertainties given by the source of the transition. Our analysis revealed that the uncertainties of about 170 transitions should be increased by one order of magnitude compared to the uncertainties declared in their sources, while the uncertainties of three transitions should be increased by two orders of magnitude. A comparison between the average claimed source uncertainty (CSU) and the average MARVEL suggested uncertainty (MSU) can be found in Table 1. This table shows that the two values, CSU and MSU, have very good overall agreement, with the exception of one source, 02Miller [42], where our analysis suggests a value one order of magnitude higher compared to the uncertainty suggested by the source.

**FIGURE 5:**
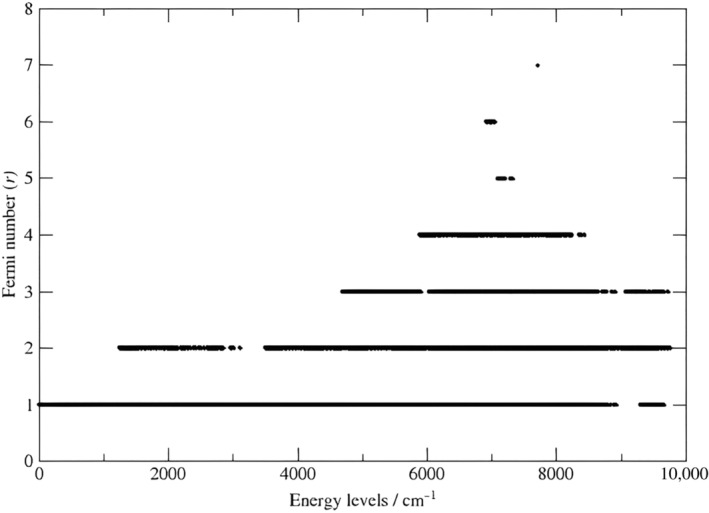
Fermi number r for each calculated energy level versus the empirical rovibrational energy levels determined in this study using MARVEL.

Figure 5 gives an idea about Fermi number r for each calculated energy level, where just 10, 7, and 1 energy levels have Fermi resonance numbers 5, 6, and 7, respectively.

### 3.2 Comparison with Other Databases

**FIGURE 6:**
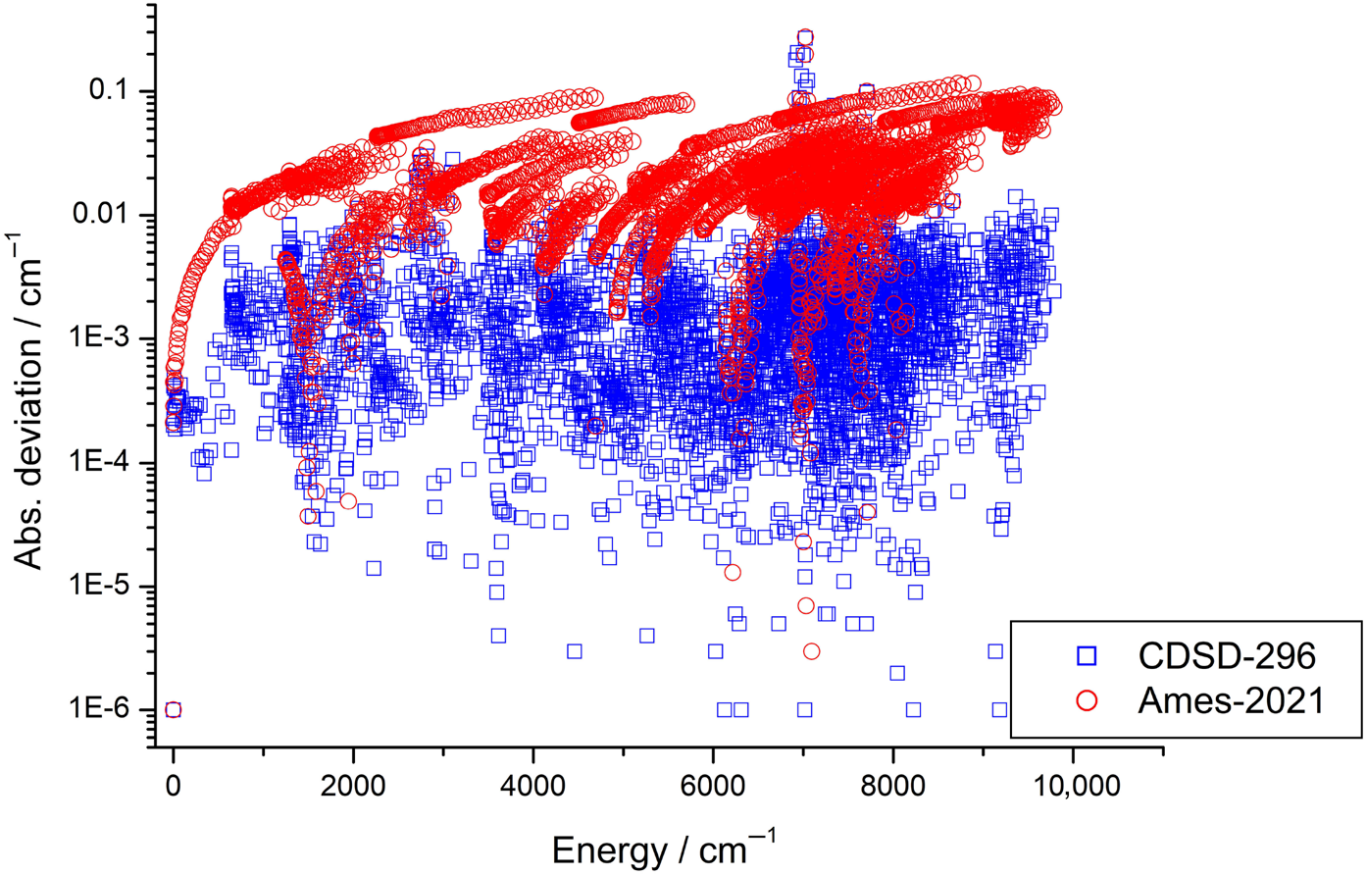
Comparison between rovibrational energies of the present ^16^O^13^C^18^O dataset and those of CDSD‐296 [12] (blue squares) and Ames‐2021 [13] (red circles).

All the empirical rovibrational energy levels reported in this study were compared with energy levels available in the databases Carbon Dioxide Spectroscopic Databank (CDSD‐296) [12] and NASA Ames‐2021 [13]. These comparisons, see Figure 6, show good overall agreement.

As seen in Figure 6, our data are in significantly better agreement with CDSD‐296, with an average deviation of only 0.002 cm^−1^, than with Ames‐2021, where the average deviation is 0.024 cm^−1^. This is not surprising, as the CDSD‐296 data are semiempirical in nature. Figure 6 shows that there are some MARVEL energy levels with deviations from the CDSD‐296 results that are significantly larger than the average. We collected these energy levels, involving the sources 04DiMaRoPe [51], 07PeKaRoPe [50], and 10CaSoMoPe [53], in Table 3. Since each energy level is determined by only one source, we could not decide whether the MARVEL or the CDSD‐296 results were correct. Settling these issues requires additional measurements of transitions involving these large‐*J* quantum states.

**TABLE 3:** The set of empirical (‘MARVEL’) energy levels of this study with differences larger than 0.05 cm^−1^ compared to their CDSD‐296 (‘CDSD’) [12] counterparts.

Labels	MARVEL energy/cm^−1^	CDSD Energy/cm^−1^	Source
27 5 1 1 0 6 f	6915.7291	6915.9096	07PeKaRoPe [50]
28 5 1 1 0 6 f	6936.5410	6936.7477	07PeKaRoPe [50]
29 3 1 1 1 3 f	6957.9903	6958.0590	06PeKaRoPe [49]
29 5 1 1 0 6 f	6958.2380	6958.3287	07PeKaRoPe [50]
30 5 1 1 0 6 f	6980.5175	6980.6523	07PeKaRoPe [50]
31 5 1 1 0 6 e	7001.1083	7001.3042	07PeKaRoPe [50]
32 5 1 1 0 6 e	7024.6895	7024.9583	07PeKaRoPe [50]
32 3 1 1 1 3 e	7024.9128	7024.8047	07PeKaRoPe [50]
33 5 1 1 0 6 e	7049.2272	7049.3500	07PeKaRoPe [50]
57 3 0 0 1 2 e	7344.3153	7344.3926	04DiMaRoPe [51]
35 4 0 0 1 4 e	7681.4510	7681.3943	10CaSoMoPe [53]
36 4 0 0 1 4 e	7707.6009	7707.7004	10CaSoMoPe [53]
36 6 0 0 0 7 e	7707.8968	7707.9952	10CaSoMoPe [53]

## 4 Summary and Conclusions

We presented a comprehensive analysis of all the available high‐resolution, rovibrational transitions of the fifth most abundant isotopologue of carbon dioxide, ^16^O^13^C^18^O. For this computational analysis, the MARVEL algorithm and code [6, 7, 8] were employed. The transitions forming the basis of the MARVEL analysis were collected from 35 literature sources. The 12 362 measured transitions collected for ^16^O^13^C^18^O cover the wavenumber range of 578−9318 cm^−1^, with the polyad number, $P=2v_{1}+v_{2}+3v_{3}$, ranging from 1 to 13. The number of unique transitions this database contains is 7432. Just 21 of all the transitions collected had to be excluded from our analysis.

The 12 362 measured transitions determine 3975 empirical energy levels, extending up to 9800 cm^−1^. The average uncertainty of the levels is 0.0025 cm^−1^. Detailed comparisons with the CDSD‐296 [12] and the NASA Ames‐2021 [13] databases reveal average differences, with respect to this work, of 0.002 and 0.024 cm^−1^, respectively. Note that CO_2_ transition intensities can be computed very accurately using *ab initio* theory [13, 59, 60]; these can be combined with MARVEL energy levels, derived here, to provide highly accurate line lists [61], which can be difficult to obtain experimentally for trace species like ^16^O^13^C^18^O.

## Supporting information

Data S1.

## Data Availability

The data involved in this paper is given in the supporting information to this paper.
